# Therapie of Huckleberry

**Published:** 1892-02

**Authors:** 


### THERAPIE OF HUCKLEBERRY.

Professor Winternitz recommends a decoction of huckleberries (saccinium myrtillus) in the different forms of diarrhœa· He covers the dried berries with cold water and cooks them for two hours, stirring them up quite frequently. After the mass is syrupy-like, he separates it from the remaining berries and presses out the juice from them. He then cools the juice, after which it is ready for use. One to two teacupsful of this juice per diem is the dose.

Winternitz claims that this preparation will act beneficially in the most pernicious cases of diarrhœa.

He uses this decoction in gonorrhœa as an injection, and claims to be very successful with such treatment.—*Blœtter fur Kin. Hydrotherapie.* (*Ex.*)

The attention of our readers is called to the advertisement of H. K. Wampole & Co., which appears on page 28 of this issue. This house is one of long standing, and enjoys a reputation of the highest character. The preparations referred to, we commend specially to the notice of practitioners.

